# Co-Existing Subaortic Stenosis in a Patient With Hypertrophic Obstructive Cardiomyopathy: A Rare and Interesting Finding

**DOI:** 10.7759/cureus.11891

**Published:** 2020-12-04

**Authors:** Raja S Mushtaque, Rabia Mushtaque, Shahbano Baloch

**Affiliations:** 1 Cardiology, National Institute of Cardiovascular Diseases, Karachi, PAK; 2 Medicine, Jinnah Postgraduate Medical Centre, Karachi, PAK

**Keywords:** hocm, subaortic membrane, lvoto, ash, sam

## Abstract

Hypertrophic cardiomyopathy (HCM) is an autosomal dominant disorder leading to left ventricular outflow tract (LVOT) obstruction. It can present with chest pain, syncope, breathlessness, or it may cause sudden cardiac death in some cases. The echocardiography in most cases while cardiac CT or cardiac MRI in selected cases are the important diagnostic modalities to make the diagnosis of HCM. In this case report, we discuss a case of a young female patient previously diagnosed with HCM and presented with palpitations, chest pain, and shortness of breath. Her echocardiography revealed severe asymmetrically hypertrophied left ventricle (LV) with normal function, the systolic anterior motion of the mitral valve was present and a subvalvular aortic membrane was also seen. The CT was also performed showing severe asymmetrical hypertrophied septum and thickened trileaflet tricommissural aortic valve with no calcification or significant valvular aortic stenosis but there was a subaortic membrane (concentric only sparing anteriorly). The presence of subaortic membrane with HCM is a rare finding and it can be a diagnostic challenge and untreated cases are susceptible to progressive heart failure and worsening of the symptoms by further increasing LVOT obstruction. A thorough investigation and planning before surgical intervention is required to achieve optimal results.

## Introduction

Hypertrophic cardiomyopathy (HCM) is an autosomal dominant disorder associated with the mutation in the genes that encode for the sarcomere proteins. It is defined as cardiac hypertrophy (wall thickness ≥15 mm, in one or more left ventricle (LV) myocardial segments) that is not explained by abnormal loading conditions (e.g. hypertension) and left ventricular outflow tract obstruction greater than or equal to 30 mmHg [1]. The global prevalence is recorded as 1 in 500 [2]. The patient typically presents with exertional chest pain, breathlessness, palpitations, and syncope, while sudden death is the severe manifestation of the disease [3]. Though the HCM remains the most common cause of sudden death in athletes while pharmacologic, electrical, and surgical interventions have reduced mortality to 0.5% per year [4]. The electrocardiogram (ECG) findings may be non-specific and vary from T wave inversion to findings suggestive of left ventricular hypertrophy (LVH). While the echocardiography is more specific and reveals asymmetrical septal hypertrophy (ASH), systolic anterior motion (SAM) of the mitral valve leaflets, left ventricle outflow tract (LVOT) obstruction, and secondary mitral regurgitation [3].

In this case report, we will discuss a patient with hypertrophic cardiomyopathy who also had subvalvular aortic stenosis (SAS) which is an interesting and rarely reported finding in patients who had HCM [5]. In most cases of subvalvular aortic stenosis (also known as subaortic stenosis), there is a membrane (usually muscular) just beneath the aortic valve causing a fixed obstruction to the blood flow across the left ventricular outflow tract [6]. Our case report mentions the diagnostic findings of SAS coexisting with HCM and describes the treatment options.

## Case presentation

**Figure 1:**
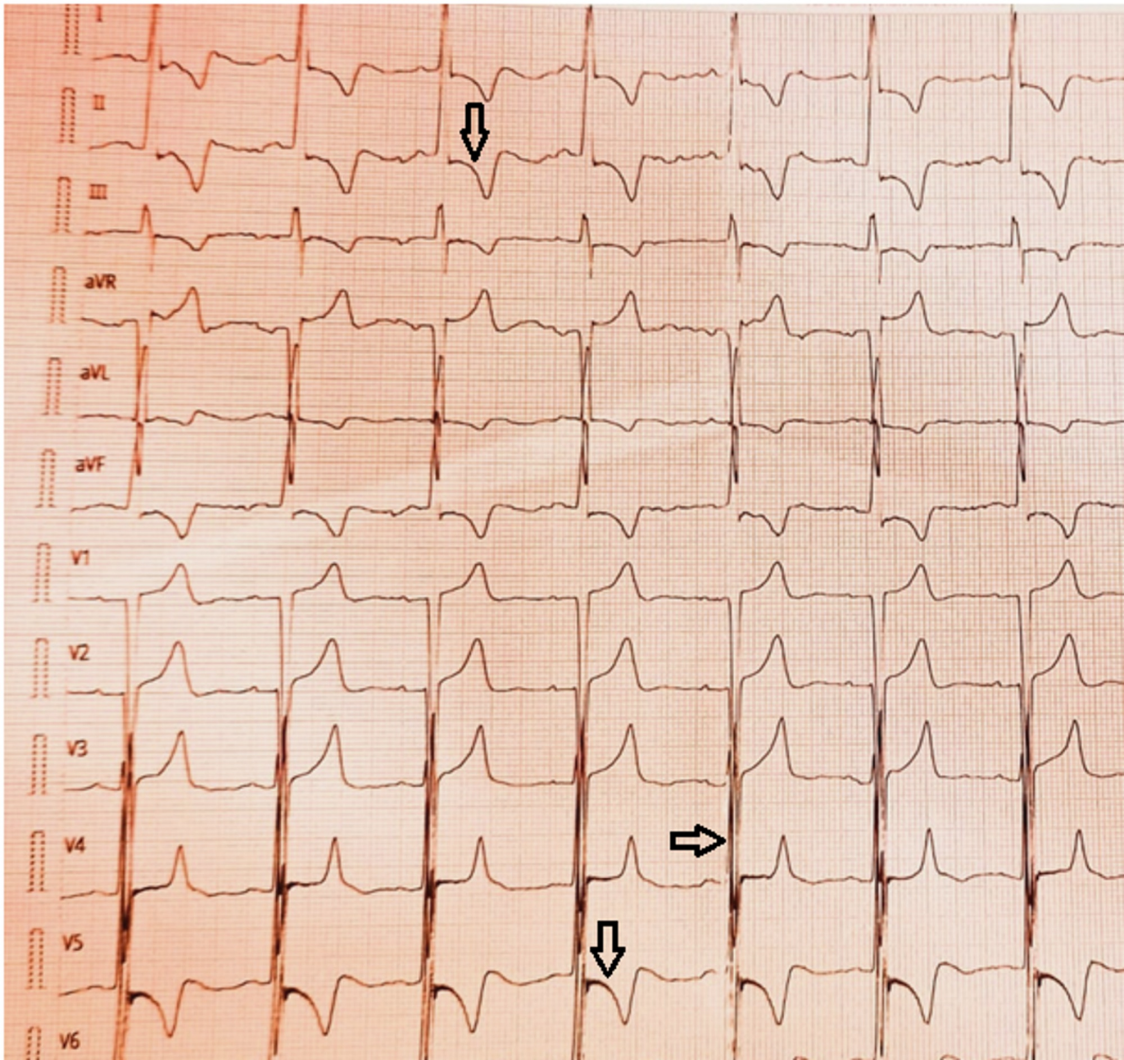
ECG of the patient shows sinus rhythm with LVH with deep T wave inversions and ST depression depicting LVH with strain pattern ECG: electrocardiogram; LVH: left ventricular hypertrophy

A 25-year-old female patient known case of hypertrophic cardiomyopathy presented with complaints of chest pain, shortness of breath, and palpitations. The patient had complained of palpitations for many years but it was exacerbated for three months. She also complained of shortness of breath for the last three months (New York Heart Association (NHYA) class III) and chest pain which is central in location and exacerbated on exertion. She denied a previous history of syncope, or any chronic medical disorder, or any drug history. She also denied any sudden death in her family. On examination, a young age female patient well oriented lying on the bed comfortably. Her blood pressure was 100/70 mmHg, pulse was 70 beats/min and regular, she was afebrile and her respiratory rate was 18/min. On her precordial examination, the apex beat was located at the fifth intercostal space lateral to the midclavicular line, and a double apical impulse was appreciated. On auscultation, S1 and S2 heart sounds were audible of equal intensity and there was ejection systolic murmur (Grade 4+) at the aortic area radiating to the carotid area. The basic laboratory workup is mentioned in Table 1. On further investigation, her electrocardiograph (Figure 1) showed sinus rhythm and left ventricular hypertrophy with deep T wave inversions and ST depression depicting LVH with strain pattern. The echocardiography revealed severe asymmetrically hypertrophied left ventricle with normal function and the systolic anterior motion of the mitral valve and dynamic left ventricular outflow tract obstruction. The aortic valve was thickened with no calcification and there was also a subvalvular aortic membrane seen. The specific findings of echocardiography are discussed in Table 2. The cardiac computed tomography showed a thickened trileaflet tricommissural aortic valve with no calcification on leaflets and aorta with no significant valvular aortic stenosis. There was severe asymmetrical hypertrophied LV, and diverticulum at mid muscular septum bulging of the membranous part of the interventricular septum (IVS) towards the right ventricle (RV) and a subaortic membrane (concentric only sparing anteriorly) resulting in LVOT obstruction, the Video 1 given below shows the details of the scan.

**Table 1:** Baseline laboratory work-up Hb: hemoglobin; MCV: mean corpuscular volume; TLC: total leukocyte count; Cr: creatinine; Na: sodium; K: potassium; PT: prothrombin time; INR: international normalization ratio; ALT: alanine transaminase; AST: aspartate aminotransferase; GGT: gamma-glutamyltransferase; ALP: alkaline phosphatase

Laboratory Investigations	Results	Normal Values
Hb	12.8	14.0-17.4 g/dl
MCV	78.2	76.5-96 fl
TLC	12.3	5.00-10.00 × 10 × 9/L
Neutrophils	62%	50-75%
Lymphocytes	32%	25-40%
Platelets	305	140-400 × 10 × 9/L
Urea	20	10-50 mg/dl
Cr	0.6	0.5-1.2
Na	137	136-149 mEq/L
K	4.2	3.50-5.50 mEq/L
Troponin I	2.67	0.0572 ng/ml
PT	12.8	9.3-14.0 seconds
INR	1.1	0.8–1.2
ALT	26	10-35 U/L
AST	21	0-31 U/L
GGT	22	0-31 U/L
ALP	71	46-116 U/L

**Table 2:** Specific dimensions given in echocardiography mm: millimeter; LVEF: left ventricle ejection fraction; LA: left atrium; LV: left ventricle; RV: right ventricle; LVOT PG: left ventricle outflow tract pressure gradient

Septal thickness (normal 11 mm)	24 mm
Posterior wall thickness (normal <11 mm)	21 mm
LVEF (>55%)	65%
Aorta (normal <40 mm)	28 mm
LA (normal <40 mm)	30 mm
LV systolic (normal <34 mm)	26 mm
RV (normal <25 mm)	31 mm
LVOT PG	140 mmHg

**Video 1:** Cardiac computerized tomography of the patient with HCM and subaortic stenosis HCM: hypertrophic cardiomyopathy

The case of this patient was discussed with the heart team and it was decided to carry out left heart catheterization first and later surgical myomectomy and resection of the subaortic membrane. But the patient requested discharge and she was kept on medical therapy with beta-blockers and advised for close follow-up.

## Discussion

The patient’s clinical presentation and diagnostic findings like asymmetrical septal hypertrophy of 24 mm, the systolic anterior motion of the mitral valve, and left ventricular outflow tract obstruction dynamic obstruction on echocardiography, and left ventricular hypertrophy with strain pattern on ECG were consistent with hypertrophic cardiomyopathy. The subaortic membrane was noticed on echocardiography as well as in cardiac computed tomography. Since it is the case of coexistent dynamic LVOT obstruction from obstructive HCM and fixed obstruction from the subaortic membrane, it may have caused abnormal thickness of other segments of the left ventricle, making HCM diagnosis challenging. The subvalvular aortic membrane is an uncommon finding and it can mimic or coexist in patients with HCM [7]. One case report mentioned, the subaortic membrane mimicked valvular aortic stenosis (AS) in which a middle-aged woman with heart failure was referred for consideration of aortic valve replacement but echocardiography revealed a subaortic membrane with an increased pressure gradient, which resolved after surgical resection [8]. Thus, it is suggested that evidence of LVOT obstruction in the absence of systolic anterior motion or valvular aortic stenosis should be considered as a potential subaortic membrane until proven otherwise [7]. The subaortic membrane may remain an under-appreciated mechanism of obstruction in patients with HCM and it increases the susceptibility of progressive heart failure and worsening of the symptoms by further increasing left ventricular outflow tract obstruction [9].

The patients with LVOT obstruction can be treated with non-vasodilating beta-blockers or calcium channel blockers (if the former not tolerated or ineffective). Patients who remain symptomatic with LVOT obstruction >50 mmHg, NYHA class III-IV, and/or recurrent exertional syncope despite maximum tolerated medical therapy should be considered for invasive treatment. The main invasive methods for relieving LVOT obstruction are surgical myomectomy (Morrow procedure) or septal alcohol ablation [3]. The subaortic membrane may require surgical myomectomy for definitive relief as percutaneous alcohol ablation would be ineffective [9]. However, there is also evidence of the recurrence of the subaortic membrane after surgical intervention and the need for reoperation in various cases [10]. In our case report, the patient was advised for surgical myomectomy and resection of the membrane to decrease pressure gradient across LVOT but the patient requested discharge and opted for pharmacological therapy. The implantable cardiac defibrillator (ICD) device can be advised after sudden death risk assessment in HCM patients and ICD may be considered if a five-year risk is 4-6% while ICD should be considered if the five-year risk is >6%. [3].

Thus, diagnosing the subaortic membrane in patients with HCM requires a high index of suspicion; un-diagnosed cases may result in progressive worsening of the symptoms and severe consequences. Echocardiography and cardiac computed tomography remain helpful diagnostic modalities and in selected cases, cardiac computed tomography and cardiac MRI are also useful. Preoperative planning and identification of this rare entity are important for optimal results [11].

## Conclusions

The subaortic membrane also is known as subaortic stenosis is a rare entity that can be isolated or co-exist with HCM and cause LVOT obstruction. A patient without any systolic anterior motion of mitral valve or valvular aortic stenosis but having LVOT obstruction should be suspected for subaortic membrane until proven otherwise. Thus, severe consequences can be avoided by early identification and treating the cause. Our case report enlightens a clinical scenario in which subaortic membrane co-exists in a patient with HCM and it also shares the diagnostic findings of imaging modalities and illustrates the management options for this patient.

## References

[1] Veselka J, Anavekar NS, Charron P (2017). Hypertrophic obstructive cardiomyopathy. Lancet.

[2] Biswas A, Das S, Kapoor M, Seth S, Bhargava B, Rao VR (2015). Epidemiology of cardiomyopathy - a clinical and genetic study of hypertrophic cardiomyopathy: the EPOCH-H study. J Pract Cardiovasc Sci.

[3] Firth J (2019). Cardiology: hypertrophic cardiomyopathy. Clin Med (London).

[4] Maron BJ (2018). Clinical course and management of hypertrophic cardiomyopathy. N Engl J Med.

[5] Camilli M, Meucci MC, Del Buono MG, Ierardi C, Delogu AB, Crea F (2020). Hypertrophic cardiomyopathy and membranous subaortic stenosis: a rare, but possible association. J Cardiovasc Med.

[6] Mulla S, Siddiqui WJ (2020). Subaortic Stenosis. StatPearls.

[7] Anderson MJ, Arruda-Olson A, Gersh B, Geske J (2015). Subaortic membrane mimicking hypertrophic cardiomyopathy. BMJ Case Rep.

[8] Carr JA, Sugeng L, Weinert L, Jeevanandam V, Lang RM (2005). Subaortic membrane in the adult. Circulation.

[9] Chung KJ, Manning JA, Gramiak R (1974). Echocardiography in coexisting hypertrophic subaortic stenosis and fixed left ventricular outflow obstruction. Circulation.

[10] Van Der Linde D, Roos-Hesselink JW, Rizopoulos D (2013). Surgical outcome of discrete subaortic stenosis in adults a multicenter study. Circulation.

[11] Kannappan M, Maron BJ, Rastegar H, Pandian NG, Maron MS, Rowin EJ (2017). Underappreciated occurrence of discrete subaortic membranes producing left ventricular outflow obstruction in hypertrophic cardiomyopathy. Echocardiography.

